# Zoom-Fatigue managen: Die Belastungen des psychotherapeutischen Online-Video-Settings erkennen und einen achtsamen Umgang mit der eigenen Gesundheit pflegen

**DOI:** 10.1007/s00729-021-00183-4

**Published:** 2021-12-06

**Authors:** Andrea Legerer-Bratengeyer

**Affiliations:** Beatrixgasse 3/21, 1030 Wien, Österreich

**Keywords:** Arbeitsplatzsicherheit, Belastungen, Bildschirmarbeitsplatz, Gesundheitskompetenz, Videosetting, Selbstfürsorge, Zoom-Fatigue, Job security, Workplace 4.0, Strain, VDU work, Video calls, Self-care, Zoom-fatigue

## Abstract

Im März 2020, bedingt durch die Corona-Pandemie, hat sich die Berufsgruppe der Psychotherapeut_innen flexibel und spontan auf die Anforderung des Online-Settings als Behandlungsformat eingelassen. Es galt die Versorgung der Klient_innen unbedingt aufrecht zu halten und einen Anteil an der Gesamtgesundheit der Bevölkerung zu leisten. Durch digitale Kommunikation im Online-Video-Setting ergeben sich für Psychotherapeut_innen bisher wenig beachtete, zusätzliche Belastungen. Insbesondere Arbeitsplatzsicherheit (Bildschirmarbeitsverordnung), Achtsamkeit und Sorgsamkeit für Augen, Binokularsystem, Rücken, Schultern und Nacken sowie der Umgang mit einer neuen Form der Erschöpfung, der Zoom-Fatigue, sind von Bedeutung. Die Arbeitswelt 4.0 hat Einzug in die psychotherapeutischen Praxen gehalten und bringt neue Aspekte und Anforderungen an Selbstfürsorge und Psychohygiene.

## Abstract (en)

In March 2020 owing to the Covid-19 pandemic psychotherapists responded spontaneously and flexibly to the demands of the video call as treatment format. The continuing provision of the clients care was deemed essential as was contributing to the general health of the population. Little observed until now, the strain experienced due to the increased usage of video sessions has grown. This article focuses particularly on job security (VDU work regulation), mindfulness of and care for the eyes, the binocular system, back, shoulders and neck. The dealing with Zoom Fatigue, a new form of exhaustion, is of relevance too when considering physical health. The Workplace 4.0 has found its way into psychotherapeutic practices and brings new aspects and challenges to self-care and mental hygiene.

Im März 2020 erzwang die Corona-Pandemie eine radikale Veränderung unserer Kommunikation. Um mit anderen Menschen in Kontakt bleiben zu können und gleichzeitig eine visuelle Wahrnehmung des anderen zu erfahren, steigt die Anzahl der Videokontakte rasant. Unterschiedliche Videokonferenzsysteme kommen hierfür zum Einsatz. Ein Anbieter eines Videodienstes ist das Unternehmen *Zoom*, welches zum Namensgeber der Zoom-Fatigue geworden ist – einem Erschöpfungszustand, der unmittelbar nach einem virtuellen Meeting auftritt.

Betroffene fühlen sich müde, gereizt und frustriert. Körperliche Symptome wie Verspannungen im Nacken- und Schulterbereich sowie Augen- und Kopfschmerzen sind häufige Kennzeichen der Erschöpfung (Lehmann-Willenbrock 2021).

Frustration wird eng verknüpft mit mangelhafter Medienkompetenz (Brown Epstein 2020). Die neuen Anforderungen der Technik verunsichern, beanspruchen Energie und lenken leicht vom eigentlichen Auftrag der Begegnung im virtuellen Raum ab. Kennzeichen von Zoom-Fatigue treten bei Menschen auf, die unvorbereitet und mit einem zu hohen Anspruch (Workload) Videokonferenzsysteme in das Arbeitsleben integriert haben (Hacker et al. 2020).

Zusätzlich verursacht der Einsatz von technischen Hilfsmitteln nicht zu vernachlässigende Kosten. Viele Menschen verwenden kleine Endgeräte wie Laptops und Notebooks oder alte Computer, die ungeeignet für den Einsatz des Video-Settings sind. Eine neue Ausrüstung, die möglicherweise nach der Corona-Pandemie nicht mehr gebraucht wird, stellt einen zusätzlichen Kostenfaktor dar, der Stress verursachen kann (Reinach Wolf 2020).

Psychotherapeut_innen erleben derzeit eine nie dagewesen Doppelbelastung. Als Menschen sind sie von den globalen Geschehnissen und Maßnahmen Betroffene, im professionellen Berufsvollzug sorgen sie für Stabilität und halten die psychotherapeutische Versorgung aufrecht. Diejenigen, die sich auf das Online-Video-Setting eingelassen haben, sind zusätzlich mit den Anforderungen und Belastungen durch die vermehrte Bildschirmaktivität konfrontiert.

Eine weitere mögliche Stressquelle stellt die Konzeption der Online-Video-Sitzung dar. Während das eigentliche (online) Gespräch dem Gespräch im Praxisraum sehr ähnlich ist unterscheiden sich das „Miteinander in Kontakt kommen“ sowie der Gesprächsabschluss deutlich vom Präsenzsetting. Der Einstieg in die gemeinsame Arbeit ist durch das Zuschalten der Teilnehmenden sehr abrupt und das Ende ist gekennzeichnet durch einen Klick. Entsprechend dieser spezifischen Anforderung des Online-Video-Settings muss ein Übergang in und aus der Videosequenz für Klient_innen gestaltet werden (Engelhardt und Engels 2021; Sümmerer 2020).

Sümmerer (2020) berichtet von Hinweisen darauf, dass sich Psychotherapeut_innen verantwortlich fühlen für die Internetverbindung. Bei technischen Schwierigkeiten entstehen Schuldgefühle und verschieden Mechanismen der Kompensation kommen zur Anwendung – z. B. Verantwortungszuweisung oder besonders intensive Aufmerksamkeit.

Die Zoom-Fatigue hat weniger mit dem Inhalt des Gespräches zu tun, sondern bezieht sich auf die besonderen Rahmenbedingungen des Settings.

## Ursachen der Zoom-Fatigue

Bailenson (2021) hat vier mögliche Ursachen der Zoom-Fatigue ausfindig gemacht.

### Langanhaltende Bildfixierung auf kurze Distanz

Der Bildausschnitt in einer Videokonferenz ist, bedingt durch die Größe des Bildschirms, stark eingeschränkt. Meist ist der Körper des Gegenübers nur von den Schultern aufwärts zu sehen. Eine ganzheitliche nonverbale Kommunikation kann nicht stattfinden und die Resonanz ist auf Mimik und paraverbale Elemente, wie z. B. Atmung, Tonlage und Tonstärke, beschränkt. Oftmals sind diese Rückmeldungen zusätzlich durch schlechte Bild- und Tonqualität beeinträchtigt, was aktives Zuhören erschwert (Lehmann-Willenbrock 2021). Um den Blickkontakt zum/zur Gesprächspartner_in aufrecht zu halten ist es notwendig, direkt in die Kamera zu schauen, meist ist es ein kleiner Punkt am oberen Bildschirmrand und die Augen möglichst wenig zu bewegen. Diese Anforderung ist nicht nur für uns als soziale Wesen ungewohnt, sondern auch für das Sehsystem unnatürlich und sehr anstrengend.

Augenbeschwerden, ausgelöst durch eine übermäßige Anstrengung, wie sie die Bildschirmtätigkeit darstellt sind schon sehr lange bekannt und können in dem Begriff der *Asthenopie* zusammengefasst werden. Asthenope Beschwerden sind ein Symptomkomplex, bestehend aus Kopfschmerzen, Augenschmerzen und geröteten Augen, verschwommenem Sehen, Doppelbilder, rasche Ermüdung und Muskelverspannungen, die zu geringerer Leistungsfähigkeit im Laufe des Tages führen (Orthoptik Austria 2021). Die normale Lidschlagfrequenz während des Sprechens ist mit ca. 15 Lidschlägen pro Minute angesetzt. Bei der Bildschirmtätigkeit ist die Lidschlagfrequenz signifikant auf ca. 4 Lidschläge pro Minute reduziert und die Befeuchtung der Augen ist nicht mehr gewährleistet. Spürbar wird der Mangel an Tränenflüssigkeit an der Oberfläche des Auges, das sogenannte *Sicca-Syndrom*, durch schmerzende, trockene und gerötete Augen (Messmer 2015).

### Erhöhte kognitive Anforderungen

Die Nutzung von Videokonferenzsystemen erfordert eine Reflexion des eigenen Verhaltens. Bei der Interaktion von Angesicht zu Angesicht läuft die nonverbale Kommunikation natürlich und fließend – Psychotherapeut_innen haben in umfassenden Selbsterfahrungsprozessen ausführliche Erfahrungen über sich und den eigenen Körper gemacht. Diese Selbsterfahrung fehlt beim Einsatz des Online-Video-Settings und die zuvor jahrelang erfolgreich eingesetzte Kommunikation muss adaptiert werden. Die Anpassung an die neuen Bedingungen ist fordernd, benötigt Aufmerksamkeit und „automatische Reaktionen“ (Bailenson 2021) auf nonverbale Hinweise müssen überwunden werden.

Im Online-Video-Setting finden häufiger Unterbrechungen statt. Schon ein Blick zur Seite hat eine soziale Bedeutung und Benutzer_innen erhalten ständig nonverbale Hinweise, die im Face-to-Face Kontext eine bestimmte Bedeutung hätten, im Online-Video-Setting allerdings anders decodiert werden müssen (Bailenson 2021).

Erscheint die Videoübertragung im ersten Blick als synchron, ist bei näherer Betrachtung eine kleine Zeitverzögerung vorhanden und das menschliche Gehirn ist veranlasst, diese auszugleichen. Auch diese Anstrengung kann einen kognitiven Belastungsfaktor darstellen (Wiederhold 2020).

Lehmann-Willenbrock (2021) diskutiert eine möglicherweise reduzierte Ausschüttung von Dopamin während eines Videogesprächs. Unser Gehirn sucht nach sozialen Signalen des Gegenübers (Interpretation von Körpersprache) und schüttelt daraufhin Dopamin aus, welches uns dabei unterstützt, wach, konzentriert und aufmerksam zu sein. Fehlen diese Reize oder erfahren wir verwirrende Informationen, entsteht Stress und Adrenalin und Noradrenalin werden ausgeschüttet. Ein hoher Energieverbrauch setzt ein, der in Müdigkeit und Erschöpfung endet.

### Konfrontation mit der eigenen Erscheinung am Bildschirm

Als problematischer wird die Funktion der Selbstansicht von Zoom angeführt. Benutzer_innen sehen sich während eines Gesprächs dauerhaft mit dem eigenen Bild konfrontiert. Die Wahrnehmung des eigenen Spiegelbildes kann zum einen zu prosozialem Verhalten führen, zum andren ist die ständige Selbsteinschätzung über ein Echtzeit-Feed sehr stressig (Bailenson 2021).

Bennett et al. (2021) messen diesem Aspekt weniger Bedeutung bei und empfehlen weiterführende Studien mit einem Videokonferenzprogramm, das die Einstellung *„hide self“* anbietet.

### Einschränkung der körperlichen Mobilität

Bedingt durch die Möglichkeiten der Kamera bietet sich nur ein sehr eingeschränkter Bewegungsraum an. Einmal „online“ gegangen ist der physische Raum für Benutzer_innen von Videokonferenzsystemen sehr dezimiert und entspricht der Form eines Kegels. Im Wesentlichen bedeutet das, dass die Sitzhaltung starr ist und der Blick immer geradeaus nach vorne gerichtet ist (Bailenson 2021).

Die Dauer der Nutzung hat Einfluss auf muskuläre Überbelastung im Nacken‑, Schulter- und Lumbalbereich und die bewegungsarme Zeit wird als Risikofaktor für Zivilisationserkrankungen erkannt. Bei gleichzeitiger Anwesenheit im realen Raum findet zwischen Gesprächspartner_innen deutlich mehr Bewegung statt (Gotzmann 2019).

### Genderbedingte Faktoren

Fauville et al. (2021) ergänzen einen fünften Risikofaktor. Untersucht wurde anhand der ZEF Scale (Zoom Exhaustion & Fatigue Scale), ob Persönlichkeit, Alter, Geschlecht und Ethnie einen Prädiktor darstellen. Häufiger betroffen und stärker ausgeprägt ist die Zoom-Fatigue bei Frauen. Stärker betroffen sind introvertierte und jüngere Menschen.

## Arbeit 4.0 in der psychotherapeutischen Praxis

Spätestens durch den Einsatz von Videokonferenzsystemen in der therapeutischen Arbeit ist die digitale Transformation in den Praxen der Berufsgruppe angekommen. Die Arbeitswelt 4.0 hat Einzug gehalten.

Der Begriff *Arbeit 4.0* leitet sich von *Industrie 4.0* ab und beschreibt die Nutzung und Anwendung digitaler Kompetenzen im Rahmen von Arbeit. Im Oberbegriff *4.0* finden sich auch Konzepte und Bezeichnungen wie *New Work* oder *agile Arbeit* (Lindner et al. 2018). Digitalisierung verursacht Stress und konfrontiert die Person mit Volatilität, Unsicherheit, Komplexität und Ambiguität – den sogenannten *VUKA-Bedingungen*, die in keinem anderen Bereich rasanter fortschreiten als im Gesundheitswesen (Mierke und van Amern 2018; Knape et al. 2020; Unkrig 2020).

Die Notwendigkeit, sich mit den Anforderungen der Digitalisierung im eigenen Berufsfeld auseinanderzusetzen, ist schon lange keine Willensfrage mehr, sondern eine Bedingung für eine moderne und klientenorientierte Praxisführung. Neben den vielen positiven Aspekten der Online-Video-Settings wie z. B.: größere Lebensnähe zu Klient_innen und ein höheres Maß an Autonomie für Klient_innen im therapeutischen Prozess (Engelhardt und Engels 2021) braucht es einen klaren Blick auf mögliche gesundheitliche Risiken. Schaff (2019) berichtet von einem starken Anstieg von psychischer Belastung, chronischer Überforderung, Ermüdung, Stress und Krankmeldungen und fordert, Präventionsfaktoren in den Veränderungsprozess aufzunehmen. Hardering (2020) setzt sich mit dem Sinnerleben in der Arbeit 4.0 auseinander und identifiziert die Digitalisierung als Treiber von Fragmentierung und Beschleunigung: *„[…] die in der Arbeitswelt zu schwachen bzw. fehlenden Resonanzbeziehungen führen und damit Gefühle der Entfremdung forcieren können.“*

## Was bedeutet das nun für die Gestaltung des Arbeitsplatzes für Psychotherapeut_innen?

Eine digitale Praxis, ergänzend zum Präsenzsetting zu führen, kann eine wunderbare und sinnstiftende Arbeitswelt für Psychotherapeut_innen sein.

Lange Zeit war es, bedingt durch die österreichische Internetrichtlinie, schwierig in den unterschiedlichen Online-Settings zu arbeiten und jene Psychotherapeut_innen, die es wagten, haben sich in einem rechtlichen Graubereich bewegt. Humer et al. publizierten 2020 als ein Ergebnis einer Onlinebefragung von 1547 Psychotherapeut_innen, dass der Weg in die digitale Praxis, bedingt durch die Corona-Pandemie, als unproblematisch erlebt wurde. Österreichische Psychotherapeut_innen konnten den neuen Bereich „Psychotherapie via Internet“ rasch annehmen und umsetzten, erlebten Psychotherapie auf Distanz aber nicht ident mit Psychotherapie im face-to-face-Setting. Einzig in Bezug auf Sicherheit und Datenschutz wünschten sich die Teilnehmer_innen der Studie mehr Informationen (Humer et al. 2020).

Die Anforderungen des Online-Video-Settings an Gesundheitskompetenz, Selbstfürsorge und Psychohygiene verlangen allerdings eine Anpassung an die neuen, digitalen, Bedingungen. Genauso wie sich das Präsenzsetting nicht einfach 1:1 in das Online-Video-Setting übertragen lässt, sondern es einer klaren Konzeption der Online-Praxis bedarf, müssen auch die bewährten Mechanismen von Selbstfürsorge und Psychohygiene nachgerüstet oder ergänzt werden.

Die besonderen Rahmenbedingungen des psychotherapeutischen Praxisalltags stellen zumindest eine solide Basis dar, um die Zoom-Fatigue managen zu können. In der Studie „Zoom-Fatigue“ (*n* = 422) des Instituts für Beschäftigung und Employability Ludwigshafen (Rump und Brandt 2020) gaben rund 60 % der Teilnehmer_innen an, Zoom-Fatigue zu spüren. Ausgehend von dieser Gruppe (*n* = 251) empfinden 77,7 % der Befragten eine Begrenzung der Meetingzeit als hilfreich. Für 72,2 % ist eine Pause von 10 min zwischen den Meetings wirkungsvoll. Für 40,2 % ist es förderlich, wenn die Teilnehmeranzahl eines Meetings begrenzt wäre. 55,7 % wünschten sich humorvolle Meetings und 45,0 % der Befragten wollten in das Meeting miteinbezogen werden.

Psychotherapiesitzungen sind klar begrenzte Zeiteinheiten, geben absolute Transparenz bezüglich der Teilnehmeranzahl und üblicherweise findet zwischen den Sitzungen eine Pause von ca. 10 min statt. Aus dem Punkt „humorvolle Meetings“ und dem Wunsch miteinbezogen zu werden lässt sich schließen, dass Emotionalität und das Bedürfnis wahrgenommen zu werden auch auf Distanz vorhanden ist und die Qualität einer menschlichen Begegnung ausmachen.

Mehr als ein Drittel der Befragten (34,9 %) maßen der Anpassung des Arbeitsplatzes eine Bedeutung bei. Dieser Punkt ist für die Umsetzung des Online-Video-Settings in der psychotherapeutischen Praxis relevant, da eine zeitgemäße technische Ausstattung und ein Schreibtischarbeitsplatz bisher im Berufsalltag weniger zum Einsatz kamen.

## Gesundheitskompetenz und Arbeitsplatzgestaltung

Gesundheitskompetenz in Bezug auf Arbeit bedeutet, sich mit den Besonderheiten des Online-Video-Settings hinsichtlich des eigenen Körpers und Psyche auseinanderzusetzen und entsprechende Maßnahmen zu setzen. Die neue Arbeitsumgebung ist nun ein Schreibtisch mit einem Bildschirm, an dem oft stundenlang und nahezu unbewegt gesessen wird. Um sich unter diesen Bedingungen möglichst lange wohl zu fühlen, braucht es einen Raum mit angenehmer Temperatur, wenig Lärm und einem guten Klima. Schreibtisch und Sessel bilden eine Einheit und sollten in der Höhe anpassbar sein. Speziell der Sessel garantiert Standsicherheit, im Optimalfall durch ein fünfarmiges Fußkreuz und einer Lordosenstütze. Besonderes Augenmerk ist auf die Ausrichtung des Bildschirms zu legen. Es sollten weder auf dem Bildschirm noch auf dem Gesicht der User_in Blendungen oder Reflexionen entstehen. Demnach sollten Lichtquellen, unabhängig davon, ob es natürliches oder künstliches Licht ist, niemals von vorne direkt in das Gesicht leuchten, sondern ausnahmslos immer von der Seite kommen. Die Allgemeine Unfallversicherungsanstalt (AUVA) ist eine seriöse Quelle für Arbeitsplatzsicherheit und bietet im Merkblatt *M026-Bildschirmarbeitsplätze* ausreichend Information (AUVA 2021).

Oftmals arbeiten Psychotherapeut_innen als Ein-Personen-Unternehmer_innen (EPU) und können die Verantwortung der Arbeitsplatzgestaltung nicht an einen Arbeitgeber delegieren. Selbstverantwortung und sorgsamer Umgang mit den eigenen Ressourcen liegen dann ausschließlich in der eigenen Hand. Diese Unternehmensform hat den Vorteil eines hohen Grades an Selbstbestimmung, allerdings fallen EPUs bei klassischen Präventions- und betrieblichen Gesundheitsmaßnahmen durch den Raster. Nur ein persönliches Gesundheitsmanagement kann die eigene Arbeitskraft schützen (Janneck et al. 2019).

## Selbstfürsorge und Psychohygiene

Die Unverzichtbarkeit von Selbstfürsorge und Psychohygiene ist längst im Bewusstsein der Psychotherapeut_innen fest verankert und stellt ein Merkmal der Qualitätssicherung dar. Reddemann (2003) erklärt Selbstfürsorge folgend: *„Ich verstehe darunter einen liebevollen, wertschätzenden, achtsamen und mitfühlenden Umgang mit mir selbst und Ernstnehmen der eigenen Bedürfnisse.“*

Zentrales Element von Selbstfürsorge ist es, mit sich selbst in einer guten Beziehung zu stehen, die Signale der eigenen Seele wahrzunehmen und diesen sorgsam und achtsam zu begegnen. Es existieren viele unterschiedliche Zugänge, um sich etwas Gutes tun. Dies kann durch einen körperlichen, mentalen, sozialen oder auch spirituellen Zugang erfolgen. Selbst- und Zeitmanagement mag eine Rolle spielen, Supervision, Intervision oder ein einfacher Austausch mit Kolleg_innen kann sehr wohltuend sein. Ebenso bietet die Fähigkeit, berufliches und privates zu trennen und Grenzen zwischen der Therapeut_in und dem privaten Ich zu etablieren Schutz vor dem Ausbrennen (Gerhardinger 2020).

## Anregungen zur Selbstreflexion

Sich auf ein neues, digitales Setting einzulassen, fordert die Selbstreflexion von Psychotherapeut_innen besonders heraus.

Folgende Fragen dienen zur Reflexionsanregung: Welche Schlüsselressourcen sind vorhanden? Reicht meine Medienkompetenz aus? Ist mein PC leistungsstark und die Internetverbindung stabil? Habe ich eine, für das Gesundheitswesen zertifizierte Übertragungssoftware gewählt und kann dadurch Datensicherheit gewährleisten? Wer kann mir hilfreiche Rückmeldung zu meiner Bildschirmpräsenz geben? Kann ich mich auf die neuen Bedingungen voll einlassen oder dient das Setting zur Überbrückung? Fühle ich mich in das Setting gedrängt, um existentiell nicht den Boden zu verlieren und entsteht daraus Befangenheit? Kann ich es aushalten, dass ich das Setting nicht mehr alleine gestalte, sondern auf die Vertraulichkeit der Klient_in und des Videodienstanbieters angewiesen bin? Erlebe ich im Online-Setting möglicherweise einen Kontrollverlust? Kann ich Krisen im Online-Video-Setting managen? Wie geht es mir mit dem Tempo im Online-Video-Setting? Mache ich genug Pausen zwischen den Online-Video-Sitzungen? Wie finde ich körperlichen Ausgleich, um Verspannungen im Rücken‑, Schulter- und Nackenbereich zu lösen? Welche Augenübungen helfen mir bei der Entspannung des Binokularsystems? Könnte Meditation dem kognitiven Stress entgegenwirken? Gibt es analoge Beziehungen in meinem privaten Leben? – zu Menschen, zur Natur oder zu Tieren?

Sich auf etwas Neues und Unerprobtes einzulassen erfordert viel Mut. Mut, um es zu wagen, und Mut, um Fehler zu machen. Die Besinnung auf die eigenen fachlichen Ressourcen und die Versöhnung mit Sitzungen, die vielleicht nicht ganz perfekt gelaufen sind, können entlastend wirken.

## Ausblick

2005 forschten Reimer et al., was zur Verbesserung der Lebensqualität von Psychotherapeut_innen führen könnte. Die Erkenntnis, dass neben psychohygienischen Maßnahmen auch besser abgesicherte Berufsperspektiven zu einer Steigerung der Lebensqualität beitragen, ist derzeit aktueller denn je (Reimer, Jurkat, Vetter 2005). Probst et al. konnten 2020 belegen, dass es zu einer Steigerung des Stress-Levels bei österreichischen Psychotherapeut_innen kommt, wenn in Krisenzeiten (Corona-Pandemie) die therapeutische Tätigkeit die einzige Einnahmequelle darstellt (Probst et al. 2020).

Die bereits geforderte Ausbildung in Online-Therapie (Eichenberg 2021), eine rasche Umsetzung konstruktiver Gesetze und zeitgemäßer Rahmenbedingungen für synchrone und asynchrone Online-Therapie sowie die Möglichkeit einer dauerhaften Abrechnung der Online-Stunden durch die Sozialversicherungen könnten zur Gesundheit, Motivation und Entlastung der Berufsgruppe beitragen.
